# Refining a model of collaborative care for people with a diagnosis of bipolar, schizophrenia or other psychoses in England: a qualitative formative evaluation

**DOI:** 10.1186/s12888-018-1997-z

**Published:** 2019-01-07

**Authors:** Elina Baker, Ruth Gwernan-Jones, Nicky Britten, Maria Cox, Catherine McCabe, Ameeta Retzer, Laura Gill, Humera Plappert, Siobhan Reilly, Vanessa Pinfold, Linda Gask, Richard Byng, Max Birchwood

**Affiliations:** 1 Institute of Health Research, University of Exeter Medical School, St Luke’s Campus, Exeter, UK; 2 Health Research, Lancaster University, Furness Building, Lancaster, UK; 3 Community and Primary Care Research Group, University of Plymouth, Faculty of Medicine and Dentistry, Plymouth Science Park, Plymouth, UK; 4 Institute of Applied Health Research, University of Birmingham, Edgbaston, Birmingham, UK; 5 The McPin Foundation, 32-36 Loman Street, London, UK; 6 Centre for Primary Care research, University of Manchester, Oxford Road, Williamson Building, Manchester, UK; 7 Warwick Medical School, University of Warwick, Coventry, UK

**Keywords:** Collaborative care, Recovery, Schizophrenia, Bipolar, Psychosis, Feasibility studies, Formative evaluation

## Abstract

**Background:**

Many people diagnosed with schizophrenia, bipolar or other psychoses in England receive the majority of their healthcare from primary care. Primary care practitioners may not be well equipped to meet their needs and there is often poor communication with secondary care. Collaborative care is a promising alternative model but has not been trialled specifically with this service user group in England. Collaborative care for other mental health conditions has not been widely implemented despite evidence of its effectiveness. We carried out a formative evaluation of the PARTNERS model of collaborative care, with the aim of establishing barriers and facilitators to delivery, identifying implementation support requirements and testing the initial programme theory.

**Methods:**

The PARTNERS intervention was delivered on a small scale in three sites. Qualitative data was collected from primary and secondary care practitioners, service users and family carers, using semi-structured interviews, session recordings and tape-assisted recall. Deductive and inductive thematic analysis was carried out; themes were compared to the programme theory and used to inform an implementation support strategy.

**Results:**

Key components of the intervention that were not consistently delivered as intended were: interaction with primary care teams, the use of coaching, and supervision. Barriers and facilitators identified were related to service commitment, care partner skills, supervisor understanding and service user motivation. An implementation support strategy was developed, with researcher facilitation of communication and supervision and additional training for practitioners. Some components of the intervention were not experienced as intended; this appeared to reflect difficulties with operationalising the intervention. Analysis of data relating to the intended outcomes of the intervention indicated that the mechanisms proposed in the programme theory had operated as expected.

**Conclusions:**

Additional implementation support is likely to be required for the PARTNERS model to be delivered; the effectiveness of such support may be affected by practitioner and service user readiness to change**.** There is also a need to test the programme theory more fully. These issues will be addressed in the process evaluation of our full trial.

**Trial registration:**

ISRCTN95702682, 26 October 2017.

## Background

It has been well established that people with a diagnosis of schizophrenia or bipolar have poorer physical health and social outcomes. The factors contributing to this are multiple, reciprocally interacting and include difficulties with engagement between healthcare providers and service users, along with health related behaviours. [1, 2]. There is evidence that primary care is centrally involved with the care of people with a diagnosis of schizophrenia, bipolar or other psychoses in the UK: Reilly et al. [3] found that nearly a third were seen only in primary care and those seen in secondary care received only minimal support. These authors identified a number of obstacles to primary care practitioners supporting this group to achieve improved outcomes, including a lack of continuity of care within primary care teams and poor continuity and information exchange at the interface with secondary care [3]. Primary care practitioners also often lack the necessary time and training to effectively support people with these diagnoses in addressing their mental health needs [4–6]. Collaborative care has been identified as a potential strategy for overcoming these obstacles [5] and has been found to be effective in improving mental, physical and social functioning across a range of mental health conditions [7, 8]. However, few trials have included people with a diagnosis of schizophrenia or similar forms of psychosis and so it is not possible to draw conclusions about its effectiveness for this group [5, 8]. Further, trials that have included people with a diagnosis of bipolar, schizophrenia or other psychoses have principally taken place in the USA, with none in the UK, where the organisation and funding of healthcare provision is substantially different.

The PARTNERS2 (develoPing integrAted primaRy care for paTieNts with sERiouS mental illness) programme intends to address this gap through the development of a model of collaborative care for people with a diagnosis of bipolar, schizophrenia or other psychoses, which will be tested through a randomised controlled trial in three sites in England. The process of the development of the model and the intervention theory are described in more detail in Gwernan-Jones et al. [9]. A Cochrane review [8] of collaborative care approaches for people with severe mental illness, defined as schizophrenia or other types of schizophrenia like psychosis (e.g. schizophreniform and schizoaffective disorders), bipolar affective disorder or other psychosis, has identified 14 components of collaborative care interventions. These are listed in Table 1, alongside a description of how they are operationalised in the PARTNERS intervention.

**Table 1:** Description of collaborative care components included in the PARTNERS model

Collaborative care component	Expression in the PARTNERS model
1. An underpinning conceptual model of collaboration	Wagner’s Chronic Care Model elements: protocol-based planned care, the development of case management roles, support for patient self-management, expert consultation and decision support, shared information CHIME framework for personal recovery [11] (recovery processes to be targeted by the direct patient support component): connection, hope, identity, meaning, empowerment
2. Identification of patients: method	Eligible service users identified through screening of records against inclusion criteria
3. Identification of patients: setting	Primary and secondary care
4. Provider integration:	Specialist mental health worker (known as a care partner) is allocated from local secondary care community mental health team and based in GP surgeries.
5.Multi-disciplinary working	Care partner works alongside GPs and other primary care practitioners, under the supervision of a qualified mental health worker (from any mental health profession) based in local secondary care community mental health team, with access to consultation from psychiatrists if not available through supervision.
6. Systematic communication between providers	Care partners record information in shared records, including progress notes and care plans. Co-location supports face to face communication between care partners and primary care practitioners.
7. Case management	Care partners co-ordinate care, liaising with other providers (e.g. primary care practitioners, community mental health teams, community organisations) to ensure service users’ needs are met.
8. Study protocols / treatment algorithms	Intervention manual, describing the principles and approaches which should be adopted by care partners while responding flexibly to individual need.
9. Systematic monitoring / follow up	Regular review of service users at individually negotiated intervals, varying in intensity according to need, with a minimum of telephone contact three times a year and an expectation of more frequent face to face contact as standard. Routine use of standardised measures to monitor mental health.
10. Pharmacological intervention	No specific intervention, unless identified as a personal goal by the service user, leading to the development of individual action plans, which could include psychiatric review.
11. Psychological intervention	Care partner provides coaching to enable the service user to identify personally meaningful goals, individualised action plans and relevant resources and to become an active participant in managing their own health and wellbeing.
12. Education for mental health / primary care providers	Two-day training in the intervention as described in the manual provided to care partners and supervisors.
13. Patient education / promoting self-management	Care partner provides information and uses motivational interviewing approaches to encourage service user to identify and work towards personal goals related to improved physical health and mental wellbeing.
14. Shared decision making with patients	Care partner adopts a collaborative style of interaction with service users, engaging with them as an equal in the service of the aim of achieving service user empowerment, as specified by the CHIME framework.

As with other collaborative care approaches [5], the PARTNERS model is based on Wagner’s Chronic Care Model [10]. Additionally, and as a subtle departure from other collaborative care interventions, which tend to be focussed on specific disorders, it incorporates personal recovery processes [11] as a specific orientation to supporting service users to become more informed and active in managing their mental health, as well as ensuring care is focussed to support their priorities. Coaching was selected as the psycho-social intervention to support this recovery orientation and an approach supporting goals identified by individuals.

**Fig. 1:**
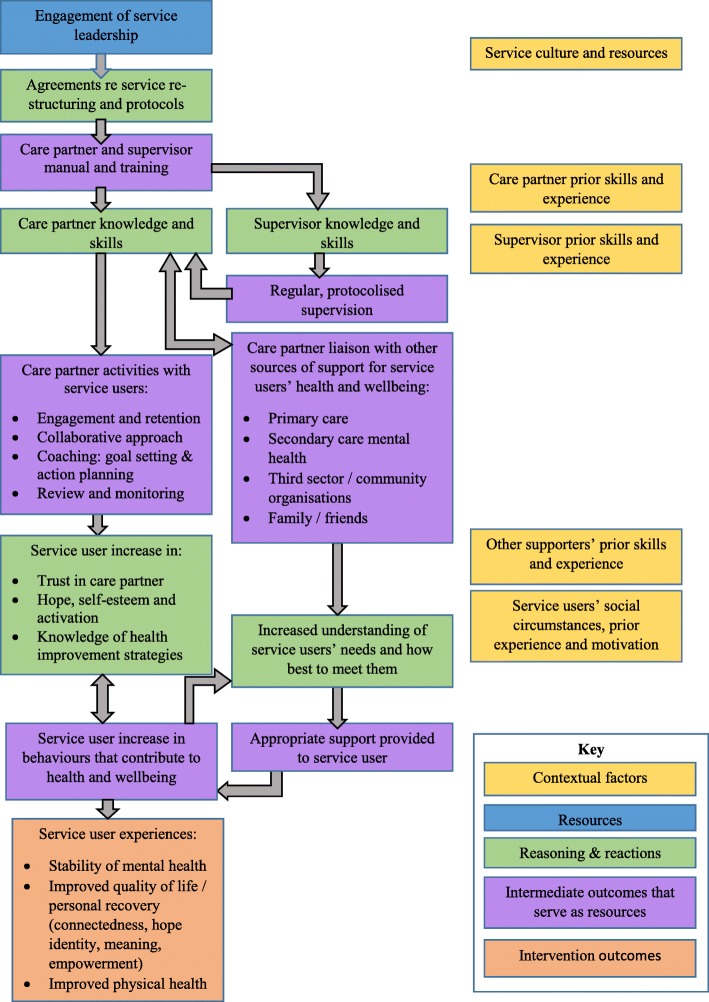
The PARTNERS2 initial programme theory

The PARTNERS programme theory was developed using realist principles [12] aiming to identify underlying causal mechanisms by which the intervention had its effects and the influence of context on the operation of these mechanisms. Within the realist approach, mechanisms are defined as the reasoning and reactions of human agents in response to the resources provided by an intervention. Mechanisms thus reflect the internal mental processes which take place within the individuals involved in an intervention that lead to them choosing to make the desired changes in practices or behaviours necessary to implement the intervention or that form the outcomes of the intervention [13]. Mechanisms include a diverse range of processes, such as increasing acceptance, motivation, knowledge and skills, trust, and self-confidence [14–16]. The initial programme theory development resulted in a large number of ‘explanatory statements’ [9], which are summarised in Fig. 1 and Table 2.

**Table 2:** Initial PARTNERS programme theory

Figure 1 represents the way in which the PARTNERS intervention operates at multiple levels with the outcomes derived from one level becoming intervention resources for the next level. In the diagram, mechanisms are broken down into the resources provided and the anticipated reasoning and reactions of the relevant actors. It is hypothesised that engagement with leadership of primary and secondary care services will lead to agreements that specialist mental health workers will be placed into primary care teams, where they will deliver care to people with a diagnosis of bipolar, schizophrenia or other psychosis who are patients of that practice, according to the PARTNERS model. These agreements are operationalised in the manual and through training delivered to care partners and supervisors. The manual and training act as resources for care partners and supervisors, supporting them to develop the knowledge and skills required to fulfil their respective roles. For supervisors, this is the provision of regular, protocolised supervision, in which they review whether the care partner is delivering the intervention as intended and provide support and guidance to ensure fidelity to the model. In turn this serves to further develop the care partners’ knowledge and skills. The care partners’ role consists of a range of activities directly with service users and communication with other people and agencies who can provide support for service users’ health and wellbeing. The support provided by care partners directly to service users is hypothesised to increase their belief in themselves and their ability to control their health and their lives, leading to an increase in service user behaviours which are likely, in turn, to lead to improved health. These include actively engaging themselves with other people and agencies who can provide support for their health and wellbeing. Successfully changing behaviour is also thought to further contribute to the service users’ confidence, creating a virtuous cycle of ongoing improvement. Care partner liaison with other sources of support is thought to lead to greater understanding in these individuals of how they can best support the service user in improving their health and wellbeing and thus the provision of support that will enable the service user to make desired changes. It is also thought to broaden care partners’ awareness of health and wellbeing needs that service users might have and the range of supports available to meet these. These mechanisms are thought to operate in contexts, which include the pre-existing characteristics of services, such as cultures and leadership style, and individuals, such as previous experience and attitudes. Thus a care partner may be more able to understand the intervention and deliver it as intended if they have previous training in coaching or a service user may be more likely to respond positively to the coaching approach if they are ready to change.	

MRC guidelines for the evaluation of complex interventions [17, 18] recommend a feasibility and piloting phase, during which formative evaluation can take place, in order to test and refine the underlying programme theory and to establish what support would be needed to ensure delivery of the intervention as intended, and thus allow effective outcome evaluation during the main trial. As implementation of collaborative care for mental health in general has been limited, despite evidence of its efficacy [19], factors affecting delivery were a particularly important focus for the formative evaluation of the PARTNERS model.

Two recent systematic reviews, which included 9 UK studies, have identified barriers and facilitators to the implementation of collaborative care for depression and anxiety [20, 21]. These included practitioners’ understanding of collaborative care and their attitudes towards change, the presence or absence of regular communication between practitioners and structures which supported this and the qualities, knowledge and skills of the case manager, in engaging both with other practitioners and service users. A potential barrier was that some service users preferred not to have mental and physical health integrated but generally the experience of service users was not investigated. These findings are consistent with other systematic reviews of factors affecting implementation of collaborative care across all conditions [22, 23] suggesting they were also likely to operate during our feasibility study. There is further some evidence that facilitation can lead to improved implementation of collaborative care models for mental health conditions [24, 25].

### Aims

In the context of the above evidence, the aims of our formative evaluation were therefore to:

1. Assess how well the intervention as delivered matched the model
2. Identify the barriers and facilitators to delivering the model as intended
3. Identify additional support for implementation likely to be required in the main trial
4. Test and refine our initial theory, through exploring any perceived effects of the intervention and comparing these to the programme theory

The use of qualitative data is recommended for addressing such aims [26] and so this methodology was adopted.

## Methods

Ethical approval was obtained from the West Midlands – Edgbaston Ethics Committee (14/WM/0052).

### Design

The formative evaluation consisted of the small scale delivery of the PARTNERS intervention in three geographically diverse regions and qualitative data collection about the experiences of the intervention from all stakeholders. In order to maximise rigour, we used multiple methods of data collection: semi-structured interviews, recordings of consultations between care partners and service users and tape-assisted recall interviews about these recordings with service users and care partners. This enabled us to triangulate multiple perspectives, including our own observations of the intervention in action, and participants’ reflections both on the intervention overall and specific interactions, allowing us to build a more detailed and accurate understanding of how the intervention had been delivered and experienced [27].

### Intervention

Through negotiations with the NHS Trusts providing secondary care mental health services in each site, agreements were reached whereby a qualified mental health worker and a senior clinician would have a portion of their time re-allocated to delivering the PARTNERS service as a care partner and supervisor respectively. The portion of time allocated varied across the sites with one Trust agreeing to a full time equivalent and another agreeing to half a day a week. The individual practitioners were nominated by the Trusts as being available for and interested in the roles. The care partners were two mental health nurses and a social worker and the supervisors were a mental health nurse, a social worker and an occupational therapist currently working as team leaders.

A number of GP (general practitioner) practices in each site were approached to establish interest in receiving the intervention. The number recruited in each site varied according to the time allocated to the care partner, with a total of six practices receiving the PARTNERS service.

To deliver the components of the intervention involving direct patient contact, care partners met individually with service users, usually at their GP practice although this was negotiated individually and some meetings took place at service users’ homes or community venues. Consultations, known as intervention sessions, varied in length according to need, preference and stage of the intervention, lasting between 20 and 90 min. Initial sessions were used to build a therapeutic relationship and develop a ‘shared understanding’ of the service user’s life experiences and priorities for change. This fed into the identification of the service user’s personal goals, the exploration of resources which could help them meet those goals and the agreement of action plans, which would be reviewed in subsequent sessions, along with routine monitoring of service users’ mental health and wellbeing using standardised measures: the CORE-10 (Clinical Outcomes in Routine Evaluation 10-item) [28] and the WEMWBS (Warwick Edinburgh Mental Wellbeing Scale) [29]. Care partners would liaise with other people and services who could provide support for the service user to work towards their goals on an individually agreed basis.

Care partners were based at GP surgeries for the allocated time with the expectation that they would become active members of that primary care team, engaging in two way consultation with other team members in whatever way was appropriate and recording information about service users in computerised record systems.

Care partners were expected to meet with supervisors at least once a fortnight for guidance on goal setting, level of intensity of care and decision-making in relation to working collaboratively with other services and agencies. A protocol was provided to ensure that care partners and supervisors maintained an overview of all service users on the caseload.

The inclusion criteria for receiving the intervention were: adults with a diagnosis of schizophrenia or bipolar, including those only receiving mental health care from general practice and those with stable but ongoing mental health needs under specialist services; individuals receiving acute crisis care were excluded. Service users were identified by screening primary and secondary care records and their suitability was confirmed by a practitioner who was known to them. They were then approached by the practitioner to discuss receiving the new service. The number of service users varied across sites, according to the time allocated to the care partner, with a total of thirty-eight service users receiving the intervention.

The intervention was delivered over a period of eight to 10 months; expectations of the minimum number of contacts a service user would receive were adjusted so that they would have at least two contacts during this period. The intervention was delivered concurrently in sites A and C but the start was delayed by 6 months in site B. No changes were made to the intervention in Site B of the basis of the evaluation of the other sites but this did influence the data collection (see below).

### Participants

Participants in the formative evaluation were service users receiving the intervention, their family carers, and practitioners involved in delivering and supporting the service. A required sample size for each category of participant in each site was agreed prior to data collection, with an aim to achieve maximum diversity in the sample rather than data saturation. This was a pragmatic decision made in relation to funding constraints and we judged that these sample sizes would provide sufficient data about the range of experiences. Data was collected from sixteen service users, five family carers, three care partners, three supervisors, four GPs and six other primary or secondary care practitioners.

### Procedure

Topic guides for semi-structured interviews were developed collaboratively by the research team, with input from members of the PARTNERS2 Lived Experience Advisory Panels (LEAPs). The LEAPs consisted of people with personal experience of living with schizophrenia or bipolar, as a service user or a family carer, and an interest in research. The study has one LEAP per site, three in total.

Topic guides focussed on the key elements of the PARTNERS service, as specified in the manual and associated service user guide, whether and how the participant had experienced these in practice and what difference this made in terms of the quality of care delivered. Time pressure on sampling prevented pilot testing of topic guides. Copies of the topic guides are available on request from the authors.

We adopted the tape-assisted recall method outlined by Cape et al., [30] whereby consultations were audio-recorded and reviewed by researchers, to identify five or six excerpts from the session where there seemed to be divergence from the model or where the care partner and service user may have perceived the care partner’s action differently. Service users and care partners were then invited to participate in separate tape-assisted recall interviews, in which these excerpts were played back and the participant was asked for their views of what had been happening at that point and whether this was helpful or unhelpful.

After service users had been receiving the intervention for a few weeks, researchers contacted them by phone to attempt to recruit them to the evaluation. Service users were asked to consent separately to the different forms of data collection. In site A, service users were sampled purposively for interviews to achieve a range of gender, diagnosis and experiences of mental health services but were identified for session recordings by the care partner. In site B, service users were identified for interviews and session recordings by the care partner on the basis of their availability and willingness to participate. In Site C, all service users were approached by researchers for interviews, although not all were available. One service user declined to have a session recorded and one was not approached for a session recording as the care partner judged it would be too distressing. Participating service users were asked to identify family carers who could be approached. Family carers were then approached by phone; all family carers who were approached agreed to be interviewed. Practitioners who had some form of contact with the PARTNERS service were contacted by phone and asked to consent to data collection relevant to their role; all those approached agreed to participate.

Data was collected over a 6 month period from sites A and C, where the intervention was delivered concurrently. Preliminary analysis of this data, which identified which components of the intervention had not been delivered as intended in these sites and possible barriers and facilitators informed the approach to data collection in site B, such that the care partner and supervisors were re-interviewed towards the end of the intervention period to see what learning through experience might have taken place.

Data was collected by a number of researchers (MC, LGa, LGi, RGJ, CMc, SR, AR), including two service user researchers. All received training in qualitative methods as appropriate, and the use of the specific interview techniques, from members of the research team experienced in qualitative research (RGJ, NB, VP, LGa).

Semi-structured interviews lasted one to 2 h and tape-assisted recall interviews lasted about 1 h. All service user and family carer interviews were conducted face to face, at the participant’s GP practice or home, according to their preference. Two service users chose to have family members present during the interview but otherwise participants were alone. Practitioner participants were interviewed face to face at their regular place of work or by telephone, according to their preference. Service user participants, care partners and supervisors had met the researcher previously as part of the process of setting up the PARTNERS service and so the interviewer would have been familiar to them. Other categories of participant had not met the interviewer previously. Service user researchers were encouraged to disclose their experience of using mental health services to service user participants. Otherwise participants were informed on Participant Information Sheets that the interviewers belonged to a group of ‘very experienced mental health researchers’. All interviews were audio-recorded and interviewers also made field notes after the interview, using an agreed template. Service users and carers received a £10 shopping voucher for each interview and recorded intervention session.

Several participants contributed to more than one source of data and the total number of data sources by type is shown in Table 3.

**Table 3:** Number and type of data sources by site

Site	Care partner interviews	Service user interviews	Supervisor interviews	Family carer interviews	GP interviews	Other primary & secondary care workers interviews	Intervention sessions	Service user tape assisted recall interviews	Care partner tape assisted recall interviews	Total
A	1	6	1	2	2	3	1	1	1	18
B	2	2	2	0	1	0	2	1	1	11
C	1	6	1	3	1	3	5	5	5	30
Total	4	14	4	5	4	6	8	7	7	59

### Data analysis

All data sources were transcribed and imported into NVivo 11. Transcripts were checked against the recordings for accuracy by researchers but not returned to participants due to time pressures created by preparing for the full trial. Preliminary deductive thematic analysis, was carried out by a number of the interviewers (MC, LGi, RGJ, CMc, AR). Data from sites A and C was coded against the detailed sub-components of the model and intended outcomes as articulated in the manual and wider programme theory. A descriptive analysis was then conducted (RGJ) that was reported and discussed at a researcher meeting, where areas for further exploration were prioritised as needing to be understood to support planning for the main trial. Written summaries of key issues relating to each component were then produced for discussion at a stakeholder meeting.

The stakeholder meeting was attended by the whole research team, care partners and supervisors and members of the LEAPs, enabling data interpretation to be grounded in lived experience. This meeting focussed on understanding why some components had not been delivered or experienced as intended and identifying strategies to address these issues; the outcome of these discussions formed the basis of the implementation support strategy and informed revisions to programme materials in advance of the full trial.

Subsequent to this meeting, further data was collected; this consisted of two tape-assisted recall interviews with the care partner and one interview with a service user in site C and all the data collection in site B. There was insufficient time to revise topic guides to take account of the emerging themes but the preliminary analysis informed the development of new topic guides for the follow up interviews with care partners and supervisors in site B. Once all the data had been collected, the entire data set was analysed by one researcher (EB) with more detailed themes, and the relationships between them, being identified and defined. Coding of data from the diverse range of sources required a degree of interpretation, especially where observational data was inconsistent with interview data or two informants gave conflicting accounts of the same event. These interpretations were supported by reference to field notes and discussion with a researcher who had been involved in collecting data and the initial coding (RGJ) and an experienced qualitative researcher (NB), taking into account the main coder’s professional background as a clinical psychologist. A framework analysis [31] was then used to systematically explore differences between data sources and reach conclusions about how best to understand the data. Interpretations were shared and discussed with the wider researcher team.

In order to address the aims of the study, the analysis initially focussed on barriers and facilitators to delivery of the intervention components. The themes identified were compared against the ongoing actions agreed at the stakeholder meeting to ensure that the emerging implementation support strategy still reflected the data. Subsequent analysis focussed on the intended outcomes of the intervention. The themes identified for each outcome domain were compared to the mechanisms hypothesised in the programme theory, as articulated in the intervention logic model and ‘explanatory statements’ [9] (see Fig. 1 and Table 2).

## Results

Sixteen service users, five family carers and sixteen practitioners participated in the formative evaluation; distribution of participants across the three sites is shown in Table 4, along with the demographic data.

**Table 4:** Distribution of participants and service user demographic data

	Service user	Family Carer	Practitioner
Site (n)			
A	6	2	7
B	3	0	3
C	7	3	6
Gender (%)			
Female	25	80	75
Male	75	20	25
Age (mean, sd)	53.3 (11.04)		
Diagnosis (%)			
Schizophrenia	44		
Bipolar	56		

We will present our findings in relation to each of our four aims, in turn

### Aim 1. Evidence of whether the intervention as delivered matched the model

The preliminary analysis identified that a number of components of the model had not been delivered as intended across the sites and one component required clearer specification as it was not clear whether what had been delivered was consistent with the intervention. The extent to which each component of the model was found to have been delivered is summarised in Table 5.

**Table 5:** Model components not consistently delivered as intended

Delivered as intended	Not delivered as intended
1. An underpinning conceptual model of collaboration	
The PARTNERS model included manualised and planned care, a case-manager, support for self-management through coaching, making specialist mental health workers readily available to primary care workers and recording in shared records. The CHIME framework was included as a specific focus of the intervention.	
2. Identification of patients: method	
Service users were identified from records and discussion with secondary care staff	
3. Identification of patients: setting	
Service users were identified in both primary and secondary care settings.	
4. Provider integration	
In two sites:	In one site:
• Care partners maintained allocated time to carry out PARTNERS role • Primary care services accommodated the care partner’s needs	• care partner required to return to secondary care role • primary care services did not give care partners access to necessary resources (e.g.: rooms, access to IT)
5.Multi-disciplinary working	
In one site:	In all sites:
• supervision took place routinely	• limited evidence of integration into primary care teams
In all sites: • access to psychiatric consultation was available	In two sites: • supervision was not delivered consistently
6. Systematic communication between providers	
In all sites:	In all sites
• a few examples of care partners making helpful entries in records, making appropriate requests to GPs and attending practice meetings	• very limited evidence of recording in shared records • very limited evidence of interaction between care partners and primary care teams
7. Case management	
In all sites:	
• evidence of care partners liaising with other providers in response to goals identified by service users or change in mental health	
8. Study protocols / treatment algorithms	
In all sites	
• care partners and supervisors were aware that the manual should guide care and evidence that they accessed the manual	
9. Systematic monitoring / follow up	
In one site:	In one site:
• repeated measures used consistently	• no evidence that repeated measures used • lack of regular follow up by care partner
In two sites: • regular follow up provided	In one site: • repeated measures used but not in a way that was consistent with the ethos of the model
	In one site: • uncertainty about whether variation in intensity could include duration as well as frequency of contact
10. Pharmacological intervention	
In all sites	
• evidence that this had been discussed as a possible personal goal and psychiatric consultation sought where relevant	
11. Psychological intervention	
In one site:	In all sites:
• coaching approach used to a large extent	• resources provided in the intervention manual to support coaching processes were rarely used
	In two sites: • very limited evidence of coaching approach being used
12. Education for mental health / primary care providers	
In all sites:	
• training provided	
13. Patient education / promoting self-management	
In one site:	In two sites:
• motivational approach used to a large extent	• very limited evidence of motivational approach being used
14. Shared decision making with patients	
In one site	In all sites:
• collaborative style of interaction largely present between care partner and service user	• service user guide intended to support service user participation not widely used
	In two sites: • very limited evidence of a collaborative style of interaction between care partners and service users

### Aim 2. Barriers and facilitators to delivering the model as intended

Further analysis initially focussed on identifying barriers and facilitators in relation to the components that had been identified as not being delivered. The most informative data was available in relation to three components: systematic communication, psychological intervention (in the form of coaching and goal setting) and multi-disciplinary working (in the form of supervision by senior mental health practitioners); we will therefore present detailed findings in relation to these.

Limited information was available about the barriers to provider integration as our topic guides did not focus on systemic issues and few of our participants worked at an organisational level where they would have been able to comment on this. Interpretation of the data in relation to the other components was complicated by inconsistencies between data sources. Data from session recordings, service user interviews and primary care practitioner interviews indicated that care partners were not fully delivering the model; however this was not necessarily recognised either by the care partner or their supervisor. Consequently care partners and supervisors did not offer any account of barriers and descriptions of facilitators were considered only partial accounts. In relation to systematic monitoring and shared decision making this prevented any firm conclusions being reached about barriers and facilitators. The themes that were identified as barriers and facilitators to systematic communication, coaching and goal setting, and supervision are shown in Table 6.

**Table 6:** Barriers and facilitators to delivery of systematic communication, coaching and supervision

Barriers	Facilitators
Systematic communication	
• Primary care service difficult to access • Care partner passive approach • Lack of support for care partner • Lack of service user interest	• Primary care service hospitable • Care partner pro-active and flexible approach • Care partner seeks support • Service user motivated to access support
Coaching and goal setting	
• Beliefs unsupportive of goal setting • Coaching incongruent with care partner style • Lack of supervision in use of coaching • Service user not motivated to change	• Goal setting valued • Coaching congruent with care partner style • Availability of supervision in use of coaching • Service users motivated to change
**Supervision**	
• Lack of supervisor availability • Lack of supervisor understanding of model • Lack of awareness of need for change in care partner’s practice	• Supervisor makes themselves available • Supervisor understands model • Awareness of need to support care partner’s development

#### Systematic communication

The extent to which care partners communicated with primary care teams appeared to be influenced both by the atmosphere of the primary care service and the communication style of the care partner. Communication was most likely to take place when the primary care service was hospitable and the care partner had an interpersonal route in to the care team, who could raise awareness of the care partner’s role and provide practical support:

*the manager…was very welcoming and introduced me to as many people as possible and… assigned a – I think she’s an admin worker or something, to me, so if I have any problems I just go to M. and M. does everything, and it’s great.* [care partner 3]

Conversely if the care partner’s presence went ‘*under the radar*’ [GP4] this acted as a barrier.

Care partners were still able to successfully communicate with primary care teams, if they were pro-active in creating and capitalising on opportunities to do so, either routinely, through using records or attending meetings, or for a specific clinical purpose. However, care partners’ lack of knowledge about primary care systems and processes could reduce their confidence to approach primary care team members, such that they focussed on more familiar clinical work rather than liaison:

*in [name of surgery] I feel that it’s kind of in process now, it’s working, people are turning up and it’s almost like, well, do I need to do something? People are aware that I’m there, but I don’t feel that they have the quality understanding of why I’m there* [care partner 3]

The support that care partners sought, and were offered, to overcome barriers to communication was a further influence, with some supervisors not being aware that this was an area of difficulty and so the issue was not addressed:

*I can’t remember him saying that there was ever a particular issue? Not one, maybe, that he brought to supervision.* [supervisor 2]

In contrast, another care partner sought support from the research team, which seemed to lead to greater engagement with primary care.

If service users wanted the care partner’s support in accessing physical health care this also created opportunities for the care partner to liaise. However some service users did not see the value in this, which acted as a further barrier to liaison:

*If I need to see me GP about health problems, I just go to me GP, I don’t involve [name of care partner].* [service user 1]

#### Coaching and goal setting

A goal-setting approach was most likely to be delivered when both care partners and service users saw the value of it, and some service users identified it as a key active ingredient of the intervention:

*the goal-oriented approach for me is the crucial factor* [service user 11]

However, there were times when both service users and care partners questioned the value of goal setting, suggesting that the emotional support provided by the sessions was as important:

*I was just thinking about sometimes allowing the sessions to just be, because whilst there is an agenda, … of the coaching… sometimes I’ve found people don’t want to necessarily be coached, but they want to come along to the sessions* [care partner 3]

This appeared to be linked to an interpretation of ‘goals’ as relating to practical achievements rather than psychological needs, which service user 11 described as ‘*airy fairy, nebulous goals’*.

The care partner’s pre-existing personal style, and whether this was congruent with a coaching approach was also influential. Where coaching was too great a contrast to the care partner’s usual practice, this tended to result in the prioritisation of their perception of the service user’s needs, especially in relation to managing risk:

*To me, me getting [name of service user] and taking her physically and saying ‘Come on, [name of service user]’ and if I have to ‘There, there’ and wrap her up in cotton wool for a little bit, I’ll know the job was done and dusted then… I’ll know if she’s safe*. [care partner 1]

The availability, or lack, of supervision in the use of a coaching approach was also linked to delivery. One supervisor was comfortable and familiar with coaching, which supported the care partner, but another was not able to provide such guidance, leaving the care partner struggling:

*I was getting meself bogged down …and I’d go to me manager and I’d say …‘ I need help here’, and it was like ‘Read your manual’ and I felt like saying [shouts] ‘You read the manual!’* [care partner 1]

Service user motivation also impacted on the delivery of coaching and goal setting, with some not engaging with the approach as they did not wish to make changes and others taking it up readily:

*some people have already set their own goals, they’ve been very good… very insightful into their own difficulties … just thinking of somebody that I’m working with at [name of surgery], she’s been very good at planning what she needs to do to improve her quality of life* [care partner 3]

#### Supervision

The delivery of systematic communication and coaching was therefore underpinned by the delivery of supervision. While some supervisors were able to prioritise providing PARTNERS supervision, in one site there were too many competing demands:

*we took on the single point of access team, recovery team as well as the crisis team, so my role sort of really expanded. So I feel really bad because I didn’t apply myself the way I probably should have done* [supervisor 1]

Consequently, the care partner felt inhibited about seeking supervision:

*I know he’s busy* [care partner 1]

The supervisor’s availability was associated with the extent to which the supervisor understood the model. When the supervisor found it hard to understand, it was more likely to be experienced as an additional demand. Conversely, regularly engaging with the model increased the supervisor’s familiarity with it and commitment to the intervention:

*the manuals are on the table but they’re closed because – I know how we’ve been working over time and I understand the model* [supervisor 3]

Whether the content of supervision was consistent with the model appeared to be influenced by the extent to which both supervisors and care partners recognised the need to challenge the care partner to engage in detailed discussions about their work. Where supervisors assumed the care partner was competent and the care partner did not recognise a need to change their practice, supervision did not focus on the intervention elements, as intended. This focus was provided when the supervisor engaged the care partner in ‘*extended case discussions*’ [supervisor 3] and the care partner valued the opportunity to reflect on their practice:

*what I like is supervisions where people are challenging me and… giving me ideas of how I can improve upon things… rather than saying ‘Oh yeah, it’s really great’* [care partner 3]

### Aim 3. Additional support for implementation likely to be required in the main trial

Drawing on these findings and approaches to facilitation that have been found to be effective in supporting the implementation of collaborative care for mental health [24, 25], we developed an implementation support strategy for the main trial. This included actions in relation to each of the three components where barriers and facilitators were identified.

#### Actions to facilitate systematic communication between care partners and primary care practitioners

- A link person to be identified within the primary care team, who will contribute to a local needs assessment, clarifying the most effective strategies for the care partner to communicate with the team about their work.
- Regular contact between researchers and care partners to ensure these strategies are being used and identify any concerns, and regular contact with the practice link person for problem-solving.
- The supervision protocol to be amended to include reviewing communication with primary care practitioners and providing support for developing communication strategies.

#### Actions to facilitate delivery of coaching and goal setting

- Revisions to care partner and supervisor manual and training, clarifying that goals can be psychological as well as practical and may include addressing the need for ongoing emotional support.
- Revisions to care partner and supervisor manual and training to provide clearer examples of coaching approaches and more opportunities for care partners and supervisors to practice through role play.
- Additional follow up training, in which care partners reflect on audio-recordings of their own work, together with supervisors to support learning from practice.

#### Actions to facilitate delivery of supervision

- Clear, negotiated agreements with secondary care service leadership about supervisors’ time commitments and regular contact between researchers and supervisors and researchers and service leadership for progress checking and trouble shooting.
- Follow up training focussed on audio recordings of care partners’ practice, to encourage critical self-refection and support supervisors engaging care partners in detailed discussion of their work.

### Aim 4. Comparison of perceived effects to the programme theory

Our preliminary analysis also identified a number of components that had been delivered but had not been experienced as intended. This mostly related to the experience of a few service users and were not universally reported. These were: not relating to the language of one of the measures used for ‘systematic monitoring’ and experiencing its use as burdensome rather than supportive; concerns about the use of coaching as the ‘psychological intervention’ because of its focus on identifying goals, which was felt to be unrealistic in the context of complex difficulties, and finding the service user guide, intended as a support for ‘shared decision making’ to be too lengthy, complex or paternalistic. Additionally, despite the provision of ‘education for mental health providers’ we found that care partners and supervisors sometimes did not have the knowledge or skills required to deliver their role in key areas following training, including physical health care and use of IT systems.

In relation to the programme theory, these findings indicate that practitioners did not react as expected to the resources provided the intervention training, in that they did not develop the knowledge required to deliver the intervention. Additionally, service users did not react as expected to some of the resources provided by the care partner, in that rather than becoming activated in managing their mental health they felt directed to attend to it in particular ways. Discussion of these findings within the team concluded that the failure of these mechanisms to operate as intended was a consequence of inadequate operationalisation of the programme theory, through the care partner manual and training and the materials used with service users, such that these did not adequately convey what was intended. It was agreed that, rather than revisions being required to the programme theory itself, revisions should be made to these practical expressions of it, in consultation with members of the LEAPs, in order to find forms of expression that would be experienced as more empowering for service users.

Further analysis focussed on the intended outcome domains shown in Fig. 1: physical health, stability of mental health and quality of life, including the personal recovery processes of connectedness, hope, identity, meaning and empowerment. The themes identified in each of these domains were compared to the programme theory, to see whether it reflected the operation of the hypothesised mechanisms.

#### Physical health

We found evidence for three mechanisms operating as predicted by the programme theory. Firstly, care partner liaison with GPs could increase GP’s understanding of service users’ needs, resulting in GPs providing appropriate support, including feeling able to attend to the service user’s physical health concerns rather than mental health:

*I can see that one patient that did come to see… my colleague, he was able to focus on his shoulder and his other thing that he’d come about and not try to do everything all at once. ‘Cos the danger is, you see a patient who’s on those sorts of drugs, you think ‘Oh god, I really need to think about their mental health’ and ‘Oh my god I need to check they’re not seeing things or they’re not in danger’, and then… there’s no time to look at stuff like stopping smoking or what their blood pressure might be* [GP 4]

Secondly, closer working with primary care could increase the care partner’s awareness of physical health issues and the support available, resulting in them introducing this as a possible goal with service users:

*it’s also having that wider perspective of kind of interest… looking at… physical history, which I wouldn’t normally have access to those kind of records* [care partner 3]

Thirdly, when the care partner shared knowledge about physical health, this could increase service user knowledge, empowering them to seek appropriate support from primary care:

*the other week she [care partner] was explaining that… I should have been having more… heart checks and stuff with the medication I’m on…and that’s the only thing that is a little bit weak, ‘cos I only had my first ever ECG [electrocardiogram]… a couple of weeks ago, and that’s ‘cos I asked for it.* [service user 12]

#### Quality of life and CHIME framework outcomes

Also consistent with the programme theory, we found evidence that service users responded to the resources provided by the care partner, particularly the coaching approach and consistency of contact, by experiencing increased hope and confidence in their ability to change as well as trust in the support from the care partner. This resulted in them taking small steps towards goals such as starting voluntary work or increasing their social contact:

*it’s giving me the confidence to get back out on the tightrope knowing that there’s safety net underneath me* [service user 11]

There was also evidence that this set up a virtuous cycle, whereby increased activity reinforced service users’ confidence, as predicted by the programme theory:

*we take the dog over there and we walk around the park a couple of times and I come home and I feel, yeah, I've got out and that's an achievement. Where beforehand I would have thought, ooh, no, no, I just can't do that.* [service user 7]

#### Stability of mental health

We also found evidence that service user responses to coaching and consistency of contact with the care partner resulted in service users changing their behaviours in ways which would help maintain the stability of their mental health, in the ways predicted. This included trust in the care partner leading to a service user becoming more open and gaining reassurance:

*there was a sense that [name of service user] felt that we were in a comfortable space, a comfortable environment for him to be able to talk about… he said that he could have bizarre, dark thoughts at times in the past. And I guess it’s a place that we can talk through those and then kind of – we can explore whether it’s something that’s out of the ordinary or whether it’s just kind of eccentric feelings, views, thoughts or whatever, to talk those things through* [care partner 3]

Additionally there was some evidence that monitoring of service users’ mental health could increase service user hope and confidence about their ability to maintain stability and access to support if necessary:

*even though I find putting a number on it is quite hard, but at the same time it gives you that general score of where you are… you might be depressed, but you're not having bad thoughts, you're not kind of not confident but you don’t need to worry* [service user 12]

There was also evidence that care partner liaison with secondary care practitioners led to increases in their understanding of individual service user needs and preferences, resulting in appropriate support being provided, in the way expected:

*links to the psychiatrist, that’s been pretty good, ‘cos there was somebody …whose medication had been changed around and as a result she’d become a lot less motivated … so I spoke with a psychiatrist here, ‘cos I knew that she was going to be meeting as an outpatient, um, to see what their thoughts were about medication, and they’ve changed it back and she’s very pleased about that* [care partner 3]

## Discussion

This formative evaluation of a novel collaborative care intervention for people with a diagnosis of bipolar, schizophrenia or other psychoses in England had four aims: we will discuss our findings in relation to each in turn.

### How well the intervention as delivered matched the model

Our data indicated that while many of the intervention components were delivered as intended, a number were only partially or inconsistently delivered, with one component requiring clearer specification as it was not clear whether it had been delivered as intended. Key components that were not delivered in at least two, if not all three, sites were: increased interaction between care partners and primary care practitioners, either through becoming integrated into primary care teams or through routinely sharing information about service users, supervision of the intervention, use of a coaching and goal-setting approach, and a collaborative style of interaction with service users.

These findings are consistent with those of other qualitative evaluations of the delivery of collaborative care for mental health in England, with limited integration of specialist mental health workers into primary care teams being most commonly identified [32–34]. Other evaluations have also identified inconsistent delivery of supervision [35] and practitioners having difficulty adapting to a new therapeutic style [36]. This indicates that these components may need additional implementation support in order to enable evaluation of the intervention in a full trial and that such support may also need to be considered if more widespread implementation is indicated.

### Barriers and facilitators to delivering the model

We identified a number of factors that influenced the delivery of increased communication, the use of coaching, and supervision: service leadership that supported organisational change through raising awareness of new roles and making resources available, the use of strategies to support regular communication between practitioners, the communication skills of care partners, both with other practitioners and service users, practitioners’ understanding of the intervention, effective supervision and service user motivation. Our results indicate that recovery-focussed collaborative care for people with a diagnosis of bipolar, schizophrenia or other psychoses is most likely to be implemented when the primary care team are receptive and the secondary care practitioner is prepared to adopt new working practices, including a pro-active approach to liaison and a more positive approach to risk. Further, that such changes to practice may be most effectively supported by supervisors with sufficient time and knowledge and when practitioners are sufficiently self-aware to engage in focussed reflection on practice. The intervention is most likely to be accepted by service users who are ready and willing to make changes, especially in addressing their physical health.

The barriers and facilitators that we identified are largely similar to those described in two recent systematic reviews of the implementation of collaborative care for depression [20, 21]. However, there has been limited previous investigation of service user views and experiences of collaborative care for mental health [21]; our findings indicate that service user motivation may also be a contributing factor. These reviews conclude that evidence-based approaches to implementation should be used to overcome such barriers, especially adequate training and supervision and setting up robust systems for communication; we have attempted to achieve this through our implementation strategy.

### Implementation support required for the main trial

Our implementation strategy aims to support increased interaction between care partners and primary care practitioners and the delivery of supervision through researcher facilitation, which has previously been found to be effective in supporting the implementation of collaborative care for mental health [24, 25]. This will identify communication strategies tailored to the local context and monitor their use, and monitor the delivery of supervision, engaging with service leadership to support the delivery of the new roles and systems where necessary.

We aim to improve care partner understanding and skills in relation to coaching, goal setting and collaborative interaction styles through additional training and clearer written information in the manual. The extent to which care partners adopted a coaching approach appeared to reflect their preferred style of practice. Clinicians’ personal style is a well-established barrier to implementation in primary care settings [37]and was also identified as influential during a randomised controlled trial of a team level pro-recovery intervention, which had a coaching component [38, 39]. The process evaluation of this trial identified that training was most effective when it involved opportunities to use the skills in practice, through role play with colleagues or initial experiences with service users, and receive direct personalised feedback. We have revised our training process to provide increased opportunities for skills practice and feedback, through role play and the use of audio recordings of sessions. Whether these strategies will be successful in influencing practitioners’ personal style is a remaining ‘key uncertainty’ [18] and will therefore be included as a specific research question in the process evaluation of the full trial.

Our data also indicated that when service users were ready and motivated for a collaborative care style intervention that they responded well. As, through its use of a coaching approach, the PARTNERS intervention focusses on building readiness for change, increased support for care partner skills development should also address barriers arising from service user motivation. Whether the reliable application of such an intervention can enable service users who are less receptive to become more engaged is an additional question that can be explored in the process evaluation of the full trial.

### Comparison to the programme theory

The partial delivery of key intervention components limited the extent to which we were able to test the programme theory. Our preliminary analysis identified instances where intervention components were delivered but not experienced as intended; these appeared to reflect difficulties with operationalising the intervention and learning from these contributed to the process of revising the written materials and training that supported intervention delivery.

Where there was evidence of the intervention being delivered as intended, the themes identified in relation to each of the intended outcome domains reflected the mechanisms proposed within the programme theory. This indicates that the programme theory itself does not require revision in advance of the full trial and provides some validation of the comprehensive theory development process outlined in Gwernan-Jones et al. [9]. However, the model requires more robust testing during the full trial, when we anticipate that increased implementation support will lead to greater fidelity and there will be thus be more opportunity to explore in depth how the intervention may have its effects.

### Strengths and limitations

In contrast to existing studies of the implementation of collaborative care for mental health, which did not collect data from all professional groups [20] or service users [21] we collected data from a wide range of sources,. We also made use of session recordings and linked tape assisted recall interviews, which deepened our understanding of interactions between care partners and service users [27]. However, there were inconsistencies between some data sources, and we did not make direct observations of how care partners related to primary care teams or supervisors. Consequently, interpretation of this data required a greater degree of inference about potential influences on implementation and uncertainty remains. Care partners and supervisors were possibly motivated to present a positive impression of the intervention rather than engage in critical reflection. This indicates a need for a different approach to understanding care partner and supervisor experience in the process evaluation of the full trial. Increased researcher facilitation may result in better rapport between researchers and practitioners and create opportunities for honest feedback. We have also revised our interview topic guides, to attempt to convey a spirit of mutual enquiry rather than evaluation.

Sampling of service users was to some extent reliant on care partners and is therefore likely to have been influenced by their perceptions of service users. This may have resulted in a positively skewed response from service users. A consistently purposive approach to sampling will be important in the main trial to enable exploration of the full range of service user experiences.

This study provides an example of the contribution of formative evaluation in the development of a complex intervention, of which few are available [40]. However as delivery of the intervention was variable, interpretations about how the intervention worked in practice in comparison to the realist programme theory and facilitators to delivery are based on limited data. Further, the initial data analysis was driven by a pragmatic need to address key issues in planning for the main trial which was imminently due to start and therefore did not involve detailed comparison between the mechanisms specified in the programme theory and the data. However, as the themes derived from our subsequent analysis were consistent with the logic model and detailed theory articulation, this provides some validation of our programme theory. Data analysis in the full trial will adopt a systematic approach to whether the mechanisms specified by the programme theory are operating.

## Conclusions

This study found that enhanced implementation support is likely to be required for the PARTNERS model to be delivered as intended; this has implications both for the full trial and for healthcare services attempting to establish collaborative care for mental health as routine practice. In particular, additional support may be required to improve understanding of, and increase practitioner confidence to implement, new working practices. Practitioner training may need to focus on practical skills development for supporting service user activation. It may be that the impact of introducing a recovery-focussed collaborative care intervention is limited by both practitioner and service user readiness to change and there is a need for a greater understanding of the contextual influences which contribute to this. This question will be addressed through the process evaluation in our main trial and has shaped our approach to data collection. Although we found promising indications that the PARTNERS model may operate as predicted by the programme theory, there is a need to test this more fully, an opportunity which the full trial will also provide.

## Abbreviations

CHIME: Connectedness, hope, identity, meaning, empowerment
CORE-10: Clinical outcomes in routine evaluation 10-item
ECG: Electrocardiogram
GP: General practitioner
LEAP: Lived experience advisory panel
PARTNERS: Developing integrAted primaRy care for paTieNts with sERiouS mental illness
WEMWBS: Warwick Edinburgh mental wellbeing scale
